# Variability of growing degree days in Poland in response to ongoing climate changes in Europe

**DOI:** 10.1007/s00484-016-1190-3

**Published:** 2016-05-24

**Authors:** Agnieszka Wypych, Agnieszka Sulikowska, Zbigniew Ustrnul, Danuta Czekierda

**Affiliations:** 1 Jagiellonian University, Kraków, Poland; 2 Institute of Meteorology and Water Management - National Research Institute, Kraków, Poland

**Keywords:** Growing degree days, Thermal conditions, Temperature variability, Poland

## Abstract

An observed increase in air temperature can lead to significant changes in the phenology of plants and, consequently, changes in agricultural production. The aim of the study was to evaluate the spatial differentiation of thermal resources in Poland and their variability during a period of changing thermal conditions in Europe. Since the variability of thermal conditions is of paramount importance for perennial crops, the study focused on apple, plum, and cherry orchard regions in Poland. The analysis was conducted for the period of 1951–2010 using air temperature daily data. Thermal resources have been defined using the growing degree days (GDD) index calculated independently for the whole year and during in frost-free season for three air temperature thresholds: 0, 5, and 10 °C, which determine the non-winter period, growing season, and the period of full plant growth, respectively. In addition, due to the high significance for perennials in particular, the incidence and intensity of frost during flowering were calculated. In this study, a detailed analysis of the spatial differentiation of thermal resources was first performed, followed by an evaluation of long-term variability and associated change patterns. The obtained results confirmed an increase in thermal resources in Poland as a consequence of the lengthening of the growing season. However, the frequency and intensity of spring frost, especially during flowering or even during ripening of plants, remain a threat to harvests in both the eastern and western parts of the country.

## Introduction

**Fig. 1:**
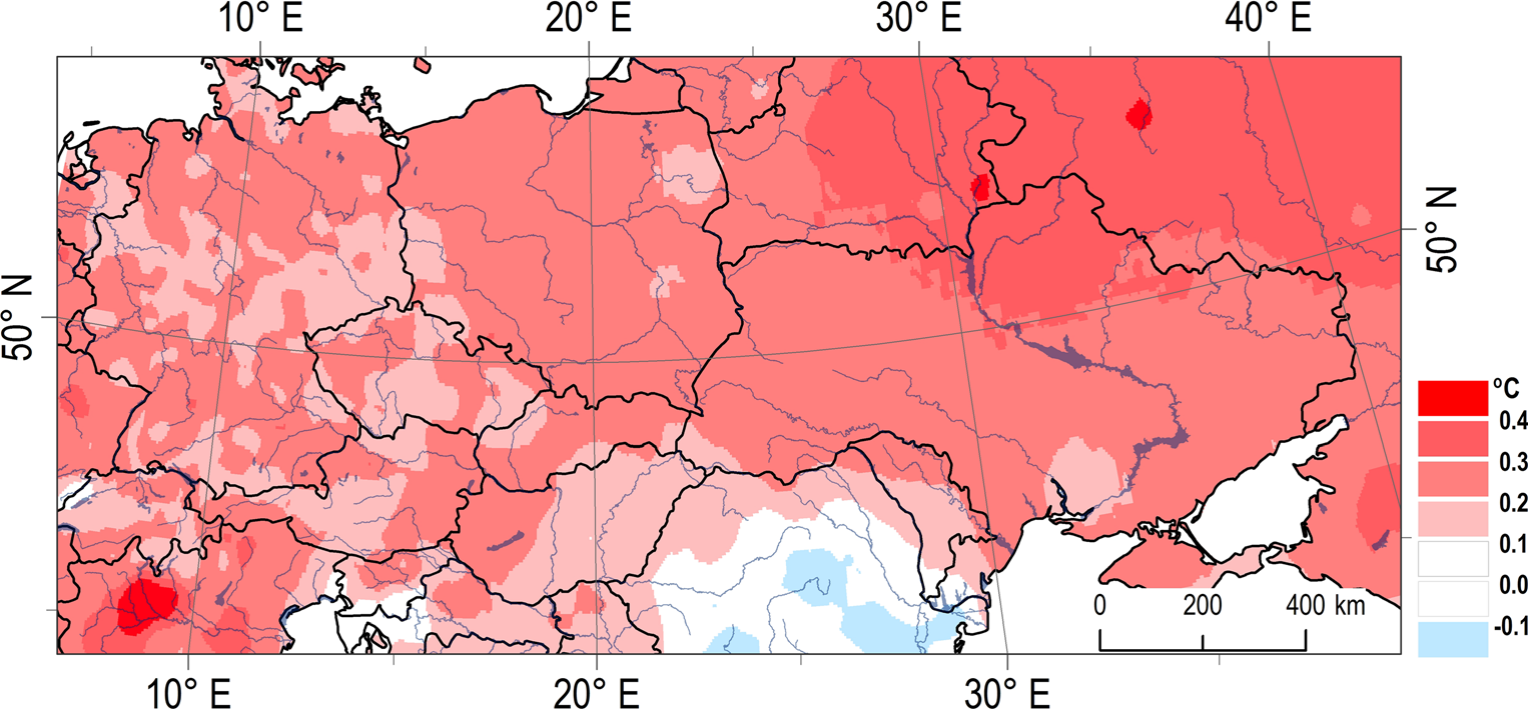
Spatial differentiation of annual air temperature tendency in Central Europe, 1951–2010 (°C/10 years) (based on E-OBS data)

The observed increase in air temperature in the northern hemisphere in recent decades is undeniable (Fig. 1) and is supported by extensive research, a summary of which can be found in the Fifth Assessment Report of the IPCC (Climate Change 2013). Due to the significant direct or even indirect effect (in interaction with other factors, such as photoperiod) of thermal conditions on the development of plants (Nyéki and Soltész 1996; Cleland et al. 2007), any change can lead to significant modifications in the phenology of plants and, consequently, also to changes in agricultural production (Żmudzka 2004; Chmielewski et al. 2004). For agriculture, horticulture, and forestry, the most important measure of thermal conditions is the length of the growing season as well as the available heat resources defined by degree day indices (Chmielewski and Rötzer 2001; Spinoni et al. 2015). Several studies have shown that the vegetation season is lengthened by approximately 5 days per 1 °C increase in the annual mean temperature (Chmielewski and Rötzer 2001) and up to 12 days per 1 °C increase in the spring mean temperature (Chmielewski and Rötzer 2001; Scheifinger et al. 2003; Menzel et al. 2006). Spring temperature tends to be largely responsible for the timing of spring phenophases (Wielgolaski 1999; Sparks et al. 2000; Menzel et al. 2006; Cook et al. 2012).

Phenological research clearly confirmed a lengthening of the growing season in the second half of the twentieth century by about 2 weeks (Climate Change 2007); this shift has accelerated in the last 3 decades (Jeong et al. 2011). While most researchers attribute this to the earlier arrival of spring (Chmielewski and Rötzer 2001; Chmielewski and Rötzer 2002; Ahas et al. 2002), the results of satellite image analyses (Jeong et al. 2011), confirming lengthening of the growing season from 1982 to 2008, show that the delay in the end of the growing season is more intense than its early onset in spring.

The mere extension of the vegetation period is not as important as the temperature rise during the growing season. This translates into an increase in heat resources, thereby speeding up or delaying the next phenophases: early budding, leafing and flowering in the spring and fruit ripening in the summer, later leaf falling in autumn (Chmielewski and Rötzer 2001).

The aim of the study was to evaluate the spatial differentiation of thermal resources in Poland and their variability in the period of 1951–2010 in the face of changing thermal conditions in Europe.

## Data and methods

**Fig. 2:**
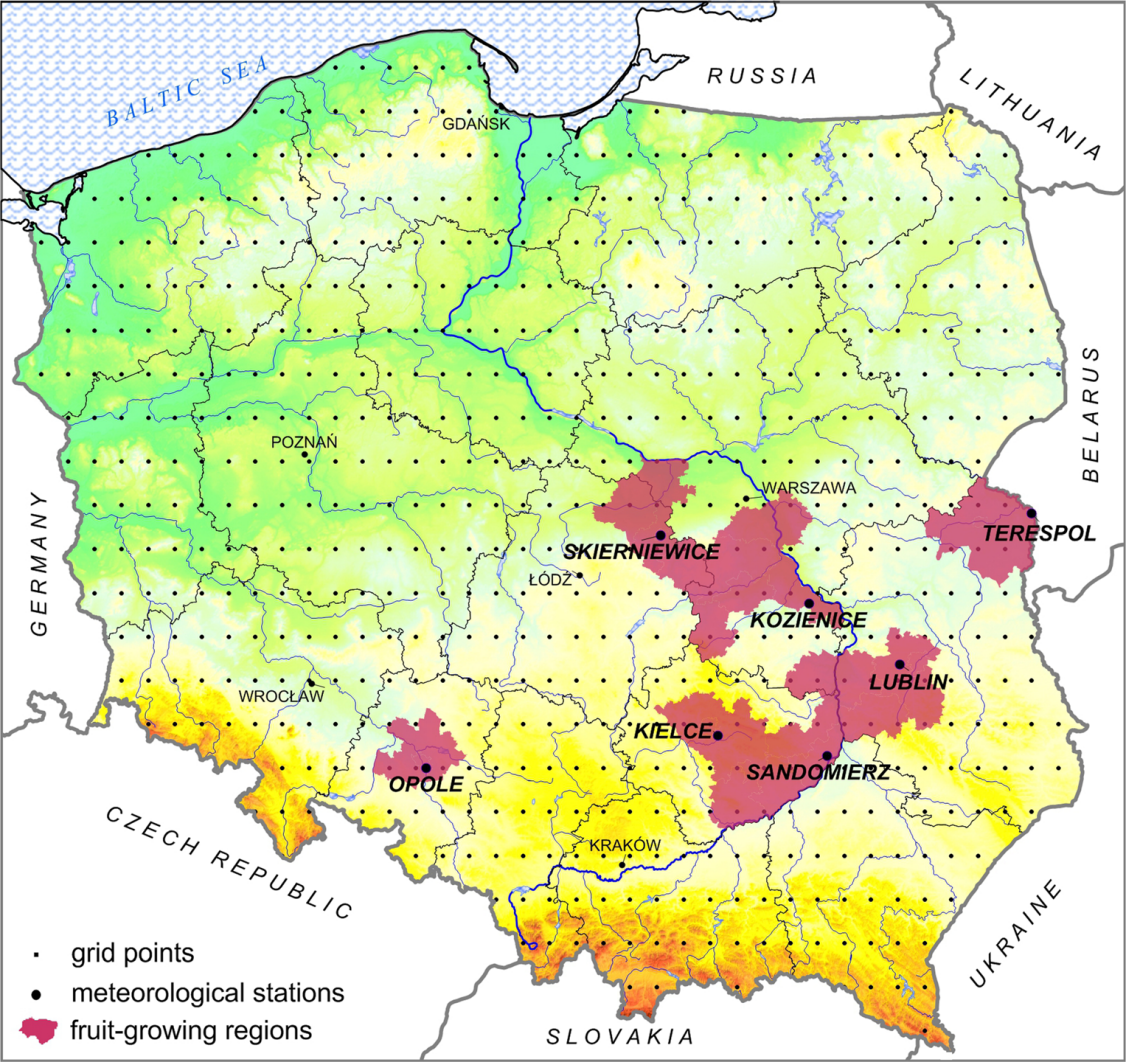
Location of the study area

Poland, whose area covers about 313,000 km^2^ and is greatly varied spatially due to its location in central Europe, seems to be a good indicator of conditions in that part of the continent. The analysis was performed for the period 1951–2010 using two independent sets of data. Daily values of average, maximum and minimum air temperature from gridded E-OBS data v.10 (Haylock et al. 2008) with a spatial resolution of 0.25° × 0.25° as well as from in situ measurements (selected meteorological stations) were used to assess the spatial differentiation of thermal resources in Poland. Since the variability of thermal conditions is of paramount importance to perennial crops, a detailed analysis was performed for the largest fruit-growing regions in the country. The regions, located respectively in the western, central and eastern parts of the country, were delimited using administrative districts with the highest orchards acreage and yields of the most productive fruits in the country, i.e., apples, plums, and cherries (Fig. 2) (Poland’s Statistical Yearbook of Agriculture 2014). An area analysis was performed for these regions using gridded data as well as detailed characteristics based on observational data. The western region is represented by the station in Opole, the central-north region by stations in Skierniewice and Kozienice, the central-south region by Kielce, Sandomierz and Lublin, and the eastern region by the station in Terespol, whereas the number of representative stations results from the size of each different region (Fig. 2). Thermal resources were defined using the growing degree days (GDD) index (1), describing the heat energy received in a given time period (McMaster and Wilhem 1997; Bonhomme 2000; Miller et al. 2001). GDD is an indicator commonly used in agro-climatology to express the amount of heat required to reach a specific phenological stage of development, which is widely used in models predicting the timeframes of individual stages of plant development (Anderson et al. 1986; Miller et al. 2001; Zavalloni et al. 2006; Matzneller et al. 2014), optimal sowing (Worthington and Hutchinson 2005), and harvesting (Łysiak 2012), as well as the threat of pests (Herms 2004; Juszczak et al. 2008).

1 $$ GDD={\displaystyle {\sum}_{i=1}^m{T}_i-{T}_{\mathrm{base}}} $$

where:

GDD: growing degree days
*T*_*i*_: daily mean air temperature [°C]
*T*_base_: threshold temperature value [°C]

GDD index values were calculated independently for the whole year and for the frost-free season; a freeze event was defined as a minimum temperature below 0 °C. Among the many applicable thresholds for *T*_base_ depending on the species, plant variety, and the purpose of the analysis itself (Yang et al. 1995; Nyéki and Soltész 1996; Snyder et al. 1999; Zavalloni et al. 2006; Matzneller et al. 2014), thresholds of 0, 5, and 10 °C, which are most frequently used for assessing the impact of thermal conditions on plant growth, were adopted as threshold temperatures, respectively, determining the non-winter period, growing season, and the period of full plant growth (Gordon and Bootsma 1993; Grigorieva et al. 2010).

As most damage to perennial fruit trees in mid-latitude locations occurs during spring bloom season when below-freezing temperatures may harm flower buds following the loss of cold hardiness (Chmielewski and Rötzer 2001; Chmielewski et al. 2004), the incidence and intensity of frost were calculated following the dates of growing degree day accumulation at the following thresholds: GDD = 150, GDD = 240, GDD = 300 (*T*_base_ = 5 °C), corresponding to early, middle, and mature bud development for the majority of fruit trees (Winkler et al. 2002).

Detailed analyses of GDD spatial distribution were followed by long-term variability and trend estimation work. The latter was performed by using a linear regression method; the *t* test was used to determine the level of statistical significance (von Storch and Zwiers 2003).

## Results and discussion

Spatial diversity and GDD trends in Europe were discussed by Spinoni et al. (2015) who strongly emphasize that in several recent decades, thermal resources have increased across the continent; changes are most pronounced in the Mediterranean basin and the weakest in the northern part of Europe. Moreover, areas located at low latitudes in the mid-twentieth century were characterized by statistically significant losses of heat resources, while positive trends have been noted only starting with the 1980s (Spinoni et al. 2015). These results are confirmed by previous studies conducted, among other places, in Russia (Grigorieva et al. 2010; Blinova and Chmielewski 2015), Poland (Żmudzka 2012), and for the Greater Baltic region (Linderholm et al. 2008).

### Spatial differentiation of growing and frost-free season length

The evaluation of heat resources in Poland was preceded by a detailed analysis of spatial differentiation of the length of growing and frost-free seasons. Exceeding the thermal threshold by approximately 5 °C, which initializes plant growth (for some plants, vegetation starts earlier, as low as 1–3 °C), thermophilic species, however, require higher air temperatures (Żmudzka 2012) followed by the occurrence of late-spring frost, which carries with it far-reaching damage to crops, especially perennial crops.

The duration of the vegetation period in Poland far exceeds the length of the frost-free season for most of the country. The biggest differences, which reach 50 to 60 days, can be seen in the southern and southwestern parts of the country (except in mountainous areas) where, with the growing season lasting more than 230 days, only 160 to 170 days are frost-free. The lowest risk of frost during growing season is noted in northern Poland, especially in coastal areas, where the difference in duration of growing and frost-free seasons does not exceed 20 days.

The number of days with frost in the spring (March–May) exceeds 35 in the mountains and exceeds 30 in the northeastern part of the country; the smallest number is noted for the coast and the southwest of Poland. Nevertheless, the last spring frosts can occur even there at the end of May or in June, with the influx of Arctic air masses from the north or northeast (Ustrnul et al. 2014).

### Spatial distribution of growing degree days (GDD)

**Fig. 3:**
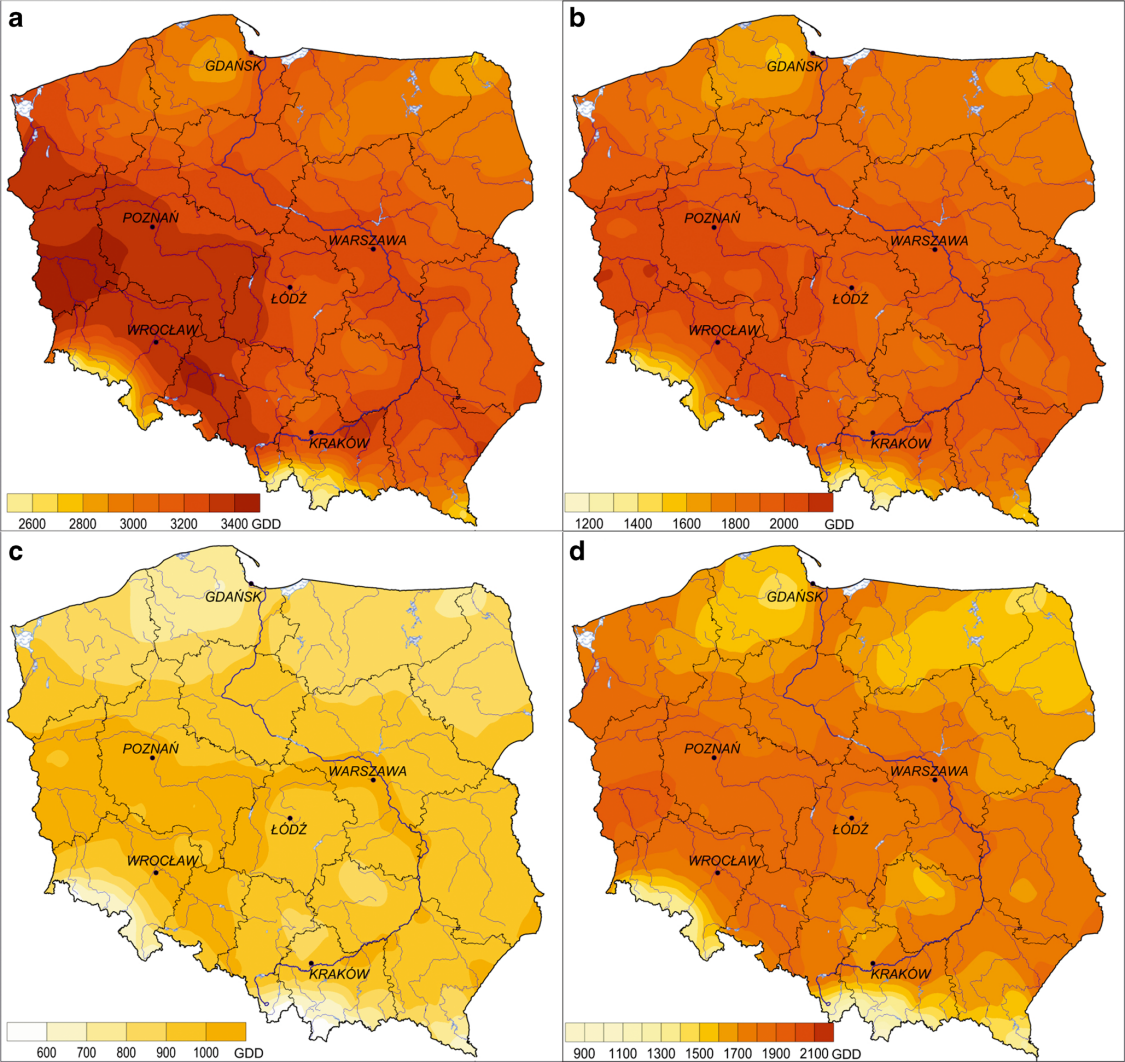
Spatial distribution of GDD in Poland. **a** *T*_base_ 0 °C. **b** *T*_base_ 5 °C. **c** *T*_base_ 10 °C. **d** Frost-free season (*T*_base_ 5 °C) (1951–2010)

Poland’s thermal resources defined on the basis of the GDD index for the years of 1951–2010 yield an average of 3150 for *T*_base_ 0 °C, 1880 for *T*_base_ 5 °C, and 920 for *T*_base_ 10 °C. Spatial diversity of GDD in Poland refers to the distribution of the average annual air temperature in the country, i.e., the regions situated in the south and southwest (except in mountainous areas) and in the valleys of the Vistula and the Oder rivers are characterized by the largest reserves of heat; fewest degree days are noted in the mountains and foothills as well as in northeastern Poland (Fig. 3). However, the regional differences, which can be seen when comparing GDD totals for different temperature bases, are worth noting. The heat totals above the threshold of 0 °C exhibit the greatest spatial diversity, reaching 1800. Geographic areas with the largest thermal resources receive slightly more than 120 % (i.e., >3800), while the areas with the least amount of thermal resources receive 65 % (i.e., approximately 2000) of the national average GDD.

In the case of the 5 and 10 °C thresholds, the range of variability is smaller (respectively, slightly less than 1500 and slightly above 1000 GDD), but their spatial distributions are much less uniform. Areas with surplus heat receive around 130 % (*T*_base_ 5 °C) and more than 150 % (*T*_base_ 10 °C) of the national average, while areas characterized by scarcity of thermal resources receive only 53 and 35 %, respectively. Such spatial distributions emphasize the variation in the occurrence of thermal seasons in Poland, including the length of the growing season, and the importance of continental influence on the eastern part of the country.

The sum of GDD values during the frost-free season (for *T*_base_ 5 °C), which is lower in areas with relatively frequent occurrence of late spring frost, confirms its important role in shaping thermal resources needed by farm crops. This situation affects more than 30 % of the country, mainly the upper catchment of the Vistula River as well as the southern and central-eastern parts of Poland.

### Long-term variability of thermal resources (GDD)

In Poland, one can observe a statistically significant change in trends in the length of both the growing season (*T*_av_ ≥ 5 °C) and the frost-free season (*T*_min_ > 0 °C), which consequently has a significant influence on changes in thermal resources. In the case of the growing season, the trend is positive across the entire country, reaching a maximum of 4 days/10 years along the Baltic coast. In contrast, the duration of the frost-free season is characterized by substantially more spatial differentiation. In southern and central Poland, its extensions (for over 5 days/10 years) have been noted. Both the earlier date of the last spring frost and the increasingly later autumn frosts occurring in the southeast are of significance here. Detailed analyses confirm that the trends described above are also affected by rising spring temperatures—primarily in March and a decreasing number of days from *T* < 0 °C for most of the area (up to 2 days/10 years in western regions). However, northeastern regions and parts of the Baltic coast are characterized by a shorter frost-free season (up to 2 days/10 years), which is associated with autumn frosts noted earlier by a maximum of 3 days.

These results are in agreement with previous studies performed for both the growing season (i.a. Żmudzka 2012; Żmudzka 2013; Graczyk and Kundzewicz 2016), and the frost-free season (i.a. Bielec-Bąkowska and Piotrowicz, 2011; Graczyk and Kundzewicz 2016), as noted at selected meteorological stations in Poland.

**Fig. 4:**
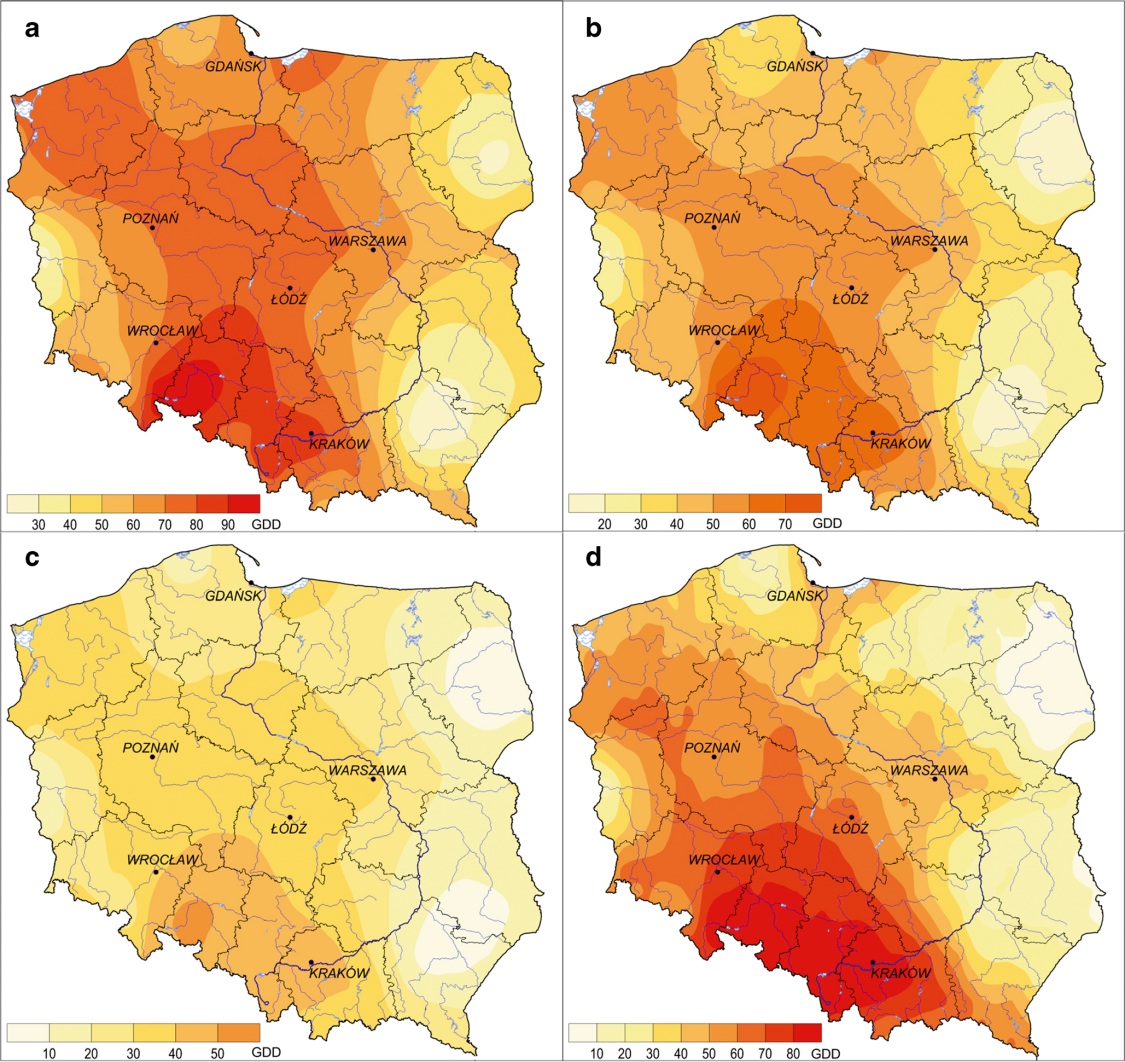
Spatial distribution of GDD tendency (per 10 years) in Poland. **a** *T*_base_ 0 °C. **b** *T*_base_ 5 °C. **c** *T*_base_ 10 °C. **d** Frost-free season (*T*_base_ 5 °C) (1951–2010)

The amount of obtained heat is closely related to the described changes during the selected characteristic time periods. The trend of thermal resources in Poland is strongly positive and statistically significant (*α* = 0.05), averaging 60 GDD for *T*_base_ 0 °C, 43 GDD for *T*_base_ 5 °C, and 28 GDD for *T*_base_ 10 °C. A preferential area in this respect is the south-central region of Poland (Fig. 4), wherein said growth reaches about 200 % compared to the average change in the country (respectively: 116, 90, and 62 GDD for the analyzed *T*_bases_). The least significant changes are observed in the eastern regions and in the middle-west of Poland (Fig. 4).

Differences in the tendencies calculated for the different thermal thresholds are worth noting and confirm the fact of increasingly important transitional seasons, especially early spring and early winter (Fig. 4a) as well as spring and autumn (Fig. 4b, d) versus the air temperature increase in summer.

### Spatial and temporal differentiation of thermal resources in fruit-growing regions

Spatial differentiation of thermal resources and associated trends observed throughout Poland determine future prospects of agricultural production in the country’s primary horticultural regions (Fig. 2).

As mentioned earlier, the differentiation in thermal resources is primarily visible for *T*_base_ 0 °C. In the analyzed horticultural regions, the GDD sums range from >3300 in the west to 3106 in the east (Table 1). In the case of the remaining thresholds, the most disadvantaged is the central-southern region of Poland. However, both these regions (i.e., eastern and central-southern) possess similar thermal characteristics.

**Table 1:** Basic characteristics of selected variables in examined fruit-growing regions (1951–2010)

Variables			Fruit-growing regions			
			Western	Central-north	Central-south	Eastern
*T*_base_ 0 °C	GDD	Average	3337	3185	3110	3106
		Minimum	2799	2693	2606	2614
		Maximum	3891	3702	3558	3506
		10-year tendency	95.0	64.7	43.7	48.2
T_base_ 5 °C	GDD	Average	1977	1894	1839	1856
		Minimum	1607	1552	1489	1503
		Maximum	2387	2308	2185	2160
		10-year tendency	65.3	42.4	27.3	27.5
T_base_ 10 °C	GDD	Average	964	924	877	904
		Minimum	720	644	594	605
		Maximum	1256	1245	1139	1168
		10-year tendency	51.0	32.3	20.4	18.8
GDD = 150	Day of a year	Average	128	128	129	129
		Minimum	114	114	116	116
		Maximum	144	146	148	148
	No. of days	10-year tendency	−1.9	−1.6	−1.3	−1.3
GDD = 240	Day of a year	Average	136	139	139	139
		Minimum	120	125	127	127
		Maximum	155	157	158	158
	No. of days	10-year tendency	−2.1	−1.7	−1.3	−1.3
GDD = 300	Day of a year	Average	143	145	147	146
		Minimum	127	131	133	133
		Maximum	162	163	166	164
	No. of days	10-year tendency	−2.2	−1.8	−1.3	−1.4

**Fig. 5:**
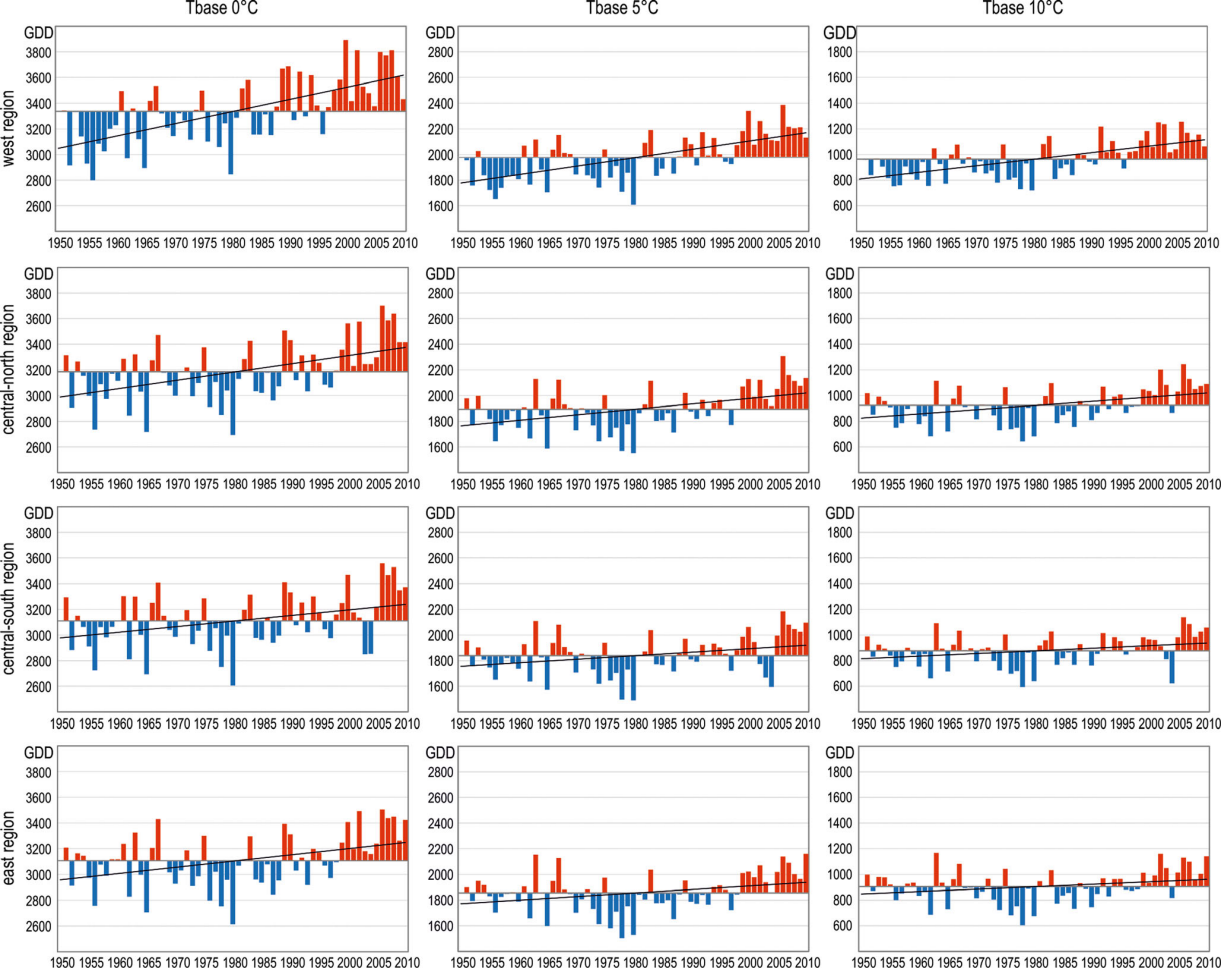
Long-term variability of GDD (at different *T*_bases_) in selected fruit-growing regions in Poland (1951–2010)

Over the long term (Fig. 5), the last few decades (since 1980) appear clearly characterized by above-average, long-term heat resources as well as a positive change trend. This undoubtedly affects the positive trend throughout the whole analyzed period, which is consistent with the results obtained by Spinoni et al. (2015) for Central Europe. GDD sums above *T*_base_ 0 °C are characterized by the greatest variability from year to year (by far), with the simultaneously largest increment for 10 years, reaching GDD of more than 90 in the western region and less than 45 in the southeast region (Fig. 5, Table 1). In the case of the remaining GDD thresholds, the observed spatial differences are equally significant, with statistically significant changes in all surveyed regions of Poland (Table 1).

Since plant growth occurs after reaching a certain amount of heat resources, the variability of the dates on which GDD totals exceeded the selected thresholds was analyzed. In this case, with statistically significant changes, spatial differences are practically negligible, especially between regions situated in the central and eastern parts of the country (Table 1). The western region of Poland is an exception, where the acceleration of subsequent phenophases reaches 2 days. Research results obtained by Jatczak and Walawender (2009) based on phenological observations conducted within the period of 1951–1992 confirm this finding.

**Fig. 6:**
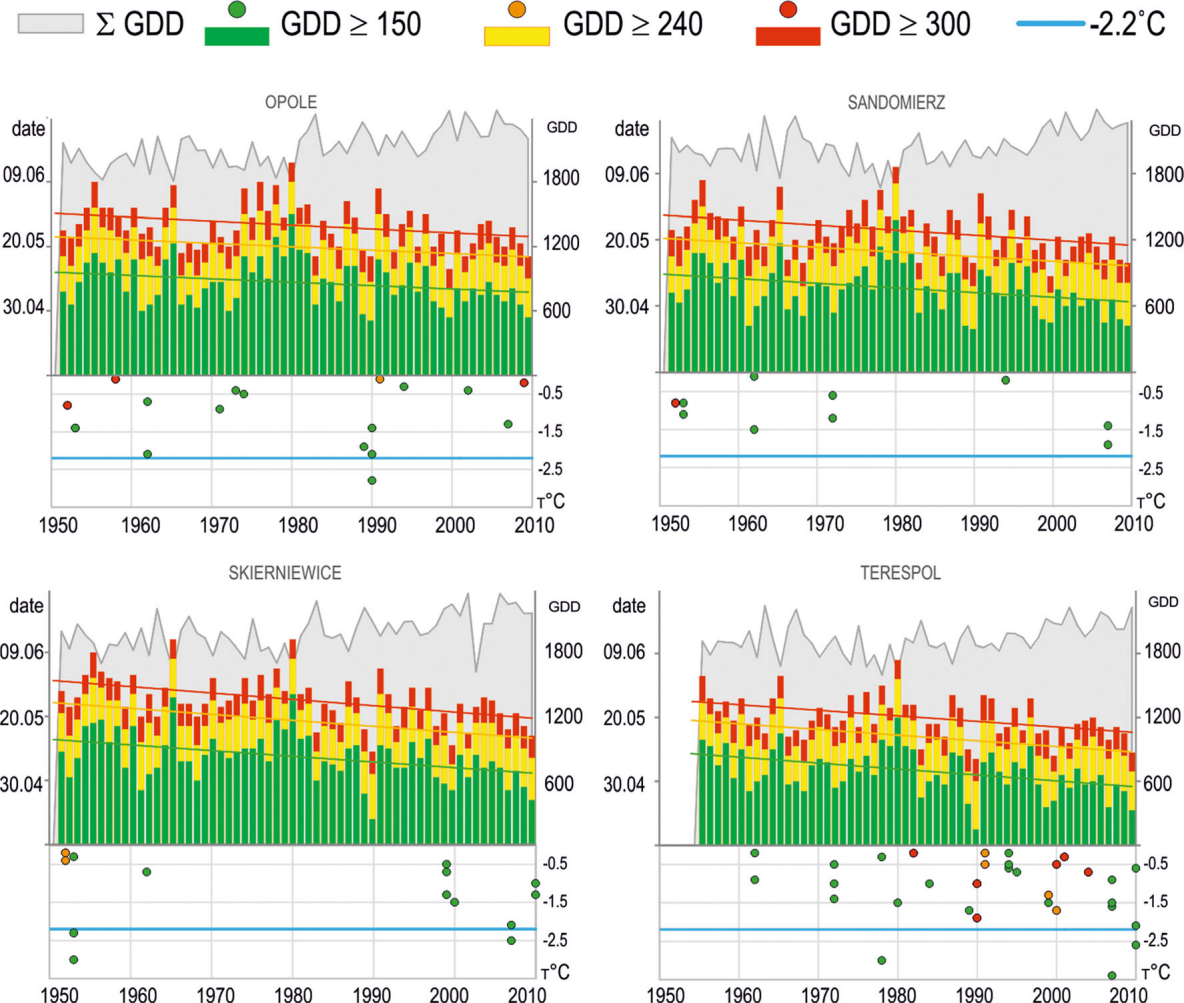
Long-term variability of GDD = 150, GDD = 240, GDD = 300 threshold dates and spring frost occurrence (after particular threshold dates) at selected stations representing fruit-growing regions in Poland (1951–2010)

Accelerated growth of plants in a transitional climate characterized by highly variable weather puts crops at risk of spring frost. In Poland, late frost can be expected even in the first 10 days of June, which is associated with an arrival of Arctic air masses from the north or northeast (Ustrnul et al. 2014). As mentioned earlier, variations in trends in the length of the frost-free season can be observed throughout the country. The surveyed horticultural regions are mostly found in areas not characterized by a trend of late spring frost, thanks to which—despite the simultaneously and previously obtained GDD thresholds (on average about 1.5 days/10 years)—the risk of frost damage to buds and flowers is limited (Fig. 6). The eastern region of Poland is under the greatest threat of frost during the successive phases of plant development. The long-term variability of thermal resources (GDD *T*_base_ 5 °C) at the station in Terespol shows an upward trend of 32 GDD/10 years (Fig. 6), with late spring frost; no statistically significant trends, aiming in the direction of extending the frost-free season. The lowest recorded air temperature was −3.4 °C after reaching the threshold of GDD = 150 and −1.9 °C for other thresholds.

In the central-north region of Poland, the upward trend associated with local heat resources is accompanied by a small, but statistically significant, change in the occurrence of spring frost. For example, in Skierniewice, frost can be expected less than 2 days in advance (Fig. 6), with a trend of GDD totals of 42.1/10 years. The lowest *T*_min_ values during the plant growth period (GDD ≥ 150) may reach −3.0 °C.

The central-southern and western regions of Poland are characterized by frost periods ending increasingly early (in both cases—Sandomierz and Opole stations—>2 days/10 years), with a significant increase in thermal resources (respectively: 29.5 and 51.9 GDD/10 years) (Fig. 6). The lowest minimum temperature during the growing season, after exceeding the threshold of GDD = 150, was −1.9 °C in Sandomierz, while in Opole, it was −2.8 °C. The lowest minimum temperature value for the remaining thresholds was −0.8 °C.

## Conclusions

Global studies on climate change impacts on agriculture, horticulture, and forestry confirm unanimously that an increase in the length of the growing season followed by a higher amount of thermal resources may enhance crop production at mid- and high latitudes, and increase potential output at harvest time as well as improve forest productivity (Winkler et al. 2002, Linderholm et al. 2008, Trnka et al. 2011). However, the rate of the observed changes may have a large impact on already living species’ distribution and the spread of insects, as well as may affect grain yields and crop seed production (Winkler et al. 2002, Thuiller et al. 2005). Moreover, the increase in heat accumulation at the time of the last spring frost event as well as the variability of the frequency of frost events after sensitive plant growth stages is reached either maintains or increases frost risk (Winkler et al. 2002, Trnka et al. 2011).

Although the research conducted so far for the territory of Poland has confirmed, as already stated, both global and regional tendencies, it was carried out only for selected stations with limited representativeness. Therefore, the results cannot be fully applied to the whole area of Poland. The use of a gridded database in the current analysis assures continuous information on temperature differentiation and provides a fairly clear picture of GDD value variability in Poland. Furthermore, it combines a thorough characterization of thermal resources across the country with frost risk analysis for several different spring phenophases expressed by GDD accumulation, with a special focus on the main fruit-growing regions of the country.

A statistically significant increase in average annual air temperature (about 0.2 °C/10 years), manifesting itself in a temperature rise in the spring months (about 0.4 °C/10 years) and in an extension of the growing season by an average of 2.5 days/10 years, has been observed. The trends described in the study result in an increase in heat resources with the greatest intensity between 0 and 5 °C (*T*_base_ 0 °C). The changes most clearly described above are observed in the southern and southwestern regions of Poland, which are considered to be warmer regions.

Future projections simulated up to 2090 give a clear image of an increase in thermal resources in Poland following changes in the length of the frost-free and growing seasons. Despite the implications of the aforesaid climate model, these thermal changes appear to be favorable for agriculture (Graczyk and Kundzewicz 2016). Taking into account the results of phenological studies confirming that an increase in air temperature by 1 °C per year accelerates the growth of plants by 2 to 5 days, and by a dozen or so days, when the increase in thermal resources concerns the spring period (i.a. Chmielewski and Rötzer 2002; Menzel et al. 2006; Jatczak and Walawender 2009), one may assume that the development of horticulture in Poland is possible with respect to the introduction of new thermophilic varieties or even new species. However, it has to be pointed out that these are hydrothermal conditions that drive crop yields and quality. Poland’s continental climate zone at middle latitudes faces significant interannual variability of precipitation amounts and water deficits, especially in the summer as a result (Trnka et al. 2011). Most of Poland features negative climatic water balance summer values (Wypych and Ustrnul 2011), which results in a decrease in water storage and summer water shortages (Szwed et al. 2010). Projected evapotranspiration intensity, driven primarily by additional temperature growth, will lead to an increasingly stressed water budget, and likely more varied or even limited rain-fed crop yields (Szwed et al. 2010, Trnka et al. 2011).

One must also remember that weather conditions, in particular the minimum air temperature as well as extreme phenomena, are largely dependent on local conditions (e.g., frost hollows) (Ustrnul et al. 2012). Mesoscale analysis for Poland and its several distinct regions, which are relatively uniform in thermal terms, though varied environmentally, does not provide a clear answer with respect to trends at the local level.

## References

[CR1] Ahas R, Aasa R, Menzel A, Fedotova VG, Scheifinger H (2002). Changes in European spring phenology. Int J Climatol.

[CR2] Anderson JL, Richardson EA, Kesner CD (1986). Validation of chill unit and flower bud phenology models for “Montmorency” sour cherry. Acta Hortic.

[CR3] Bielec-Bąkowska Z, Piotrowicz K (2011). Variability of frost-free season in Poland in the period 1951–2006. Prace i Studia Geograficzne.

[CR4] Blinova I, Chmielewski F-M (2015). Climatic warming above the Arctic circle: are there trends in timing and length of the thermal growing season in Murmansk region (Russia) between 1951 and 2012?. Int J Biometeorol.

[CR5] Bonhomme R (2000). Bases and limits to using “degree-day” units. Eur J Agron.

[CR6] Chmielewski F-M, Müller A, Bruns E (2004). Climate changes and trends in phenology of fruit trees and field crops in Germany, 1961-2000. Agric For Meteorol.

[CR7] Chmielewski F-M, Rötzer T (2001). Response of tree phenology to climate change across Europe. Agric For Meteorol.

[CR8] Chmielewski F-M, Rötzer T (2002). Annual and spatial variability of the beginning of growing season in Europe in relation to air temperature changes. Clim Res.

[CR9] Cleland EE, Chuine I, Menzel A, Mooney HA, Schwartz MD (2007). Shifting plant phenology in response to global change. Trends Ecol Evol.

[CR10] Climate Change 2007: Impacts, adaptation and vulnerability: contribution of Working Group II to the fourth assessment report of the Intergovernmental Panel on Climate Change. (2007) In: Parry, ML, Canziani, OF, Palutikof, JP, van der Linden, PJ, Hanson, CE (eds) Cambridge University Press, Cambridge

[CR11] Climate Change 2013: The Physical Science Basis. Contribution of Working Group I to the Fifth Assessment Report of the Intergovernmental Panel on Climate Change. (2013) In: Stocker TF, Qin D, Plattner G-K, Tignor M, Allen SK, Boschung J, Nauels A, Xia Y, Bex V, Midgley PM (eds) Cambridge University Press, Cambridge

[CR12] Cook BI, Wolkovich EM, Parmesan C (2012). Divergent responses to spring and winter warming drive community level flowering trends. Proc Natl Acad Sci U S A.

[CR13] Gordon R, Bootsma A (1993). Analyses of growing degree-days for agriculture in Atlantic Canada. Clim Res.

[CR14] Graczyk D, Kundzewicz Z (2016). Changes of temperature-related agroclimatic indices in Poland. Theor Appl Climatol.

[CR15] Grigorieva EA, Matzarakis A, de Freitas CR (2010). Analysis of growing degree-days as a climate impact indicator in a region with extreme annual air temperature amplitude. Clim Res.

[CR16] Haylock MR, Hofstra N, Klein Tank AMG, Klok EJ, Jones PD, New M (2008). A European daily high-resolution gridded data set of surface temperature and precipitation for 1950-2006. J Geophys Res-Atmos.

[CR17] Herms D (2004) Using degree-days and plant phenology to predict pest activity. In: Krischik V, Davidson J (eds) IPM (Integrated Pest Management) of Midwest Landscapes, Minnesota Agricultural Experiment Station Publication 58-07645, p 49–59. St. Paul

[CR18] Jatczak K, Walawender J (2009). Average rate of phenological changes in Poland according to climatic changes—evaluation and mapping. Adv Sci Res.

[CR19] Jeong S-J, Ho C-H, Gim H-J, Brown ME (2011). Phenology shifts at start vs. end of growing season in temperate vegetation over the Northern Hemisphere for the period 1982-2008. Glob Chang Biol.

[CR20] Juszczak R, Leśny J, Olejnik J (2008). Cumulative degree-days values as an element of agrometeorological forecast of the Wielkopolska region internet based agrometeorological information service (WISIA). Acta Agrophysica.

[CR21] Linderholm HW, Walther A, Chen D (2008). Twentieth-century trends in the thermal growing season in the Greater Baltic Area. Clim Change.

[CR22] Łysiak G (2012). The sum of active temperatures as a method of determining the optimum harvest date of ‘Sampion’ and ‘Ligol’ apple cultivars. Acta Scientiarum Polonorum-Hortorum Cultus.

[CR23] Matzneller P, Blümel K, Chmielewski F-M (2014). Models for the beginning of sour cherry blossom. Int J Biometeorol.

[CR24] McMaster GS, Wilhem WW (1997). Growing degree days: one equation, two interpretations. Agric For Meteorol.

[CR25] Menzel A, Sparks TH, Estrella N, Koch E, Aasa A, Ahas R, Alm-Kübler K, Bissolli P, Braslavská O, Briede A, Chmielewski F-M, Crepinsek Z, Curnel Y, Dahl Å, Defila C, Donnelly A, Filella Y, Jatczak K, Måge F, Mestre A, Nordli Ø, Peñuelas J, Pirinen P, Remišová V, Scheifinger H, Striz M, Susnik A, van Vliet AJH, Wielgolaski F-E, Zach S, Zust A (2006). European phenological response to climate change matches the warming pattern. Glob Change Biol.

[CR26] Miller P, Lanier W, Brandt S (2001) Using growing degree days to predict plant stages. Montana State University Extension Service MT00103 AG 7/2001

[CR27] Nyéki J, Soltész M (1996). Floral biology of temperate zone fruit trees and small fruits.

[CR28] Poland’s Statistical Yearbook of Agriculture (2014) http://stat.gov.pl/

[CR29] Scheifinger H, Menzel A, Koch E, Peter C (2003). Trends of spring time frost events and phenological dates in Central Europe. Theor Appl Climatol.

[CR30] Snyder RL, Spano D, Cesaraccio C, Duce P (1999). Determining degree-day thresholds from field observations. Int J Biometeorol.

[CR31] Sparks TH, Jeffree EP, Jeffree CE (2000). An examination of the relationship between flowering times and temperature at the national scale using long-term phenological records from the UK. Int J Biometeorol.

[CR32] Spinoni J, Vogt J, Barbosa P (2015). European degree-day climatologies and trends for the period 1951-2011. Int J Climatol.

[CR33] Szwed M, Karg G, Pińskwar I, Radziejewski M, Graczyk D, Kędziora A, Kundzewicz ZW (2010). Climate change and its effect on agriculture, water resources and human health sectors in Poland. Nat Hazards Earth Syst. Sci..

[CR34] Thuiller W, Lavorel S, Araújo MB, Sykes MT, Prentice IC (2005). Climate change threats to plant diversity in Europe. Proc Natl Acad Sci USA.

[CR35] Trnka M, Olesen JE, Kersebaum KC, Skjelvåg AO, Eitzinger J, Seguin B, Peltonen-Sainio P, Rötter R, Iglesias A, Orlandini S, Dubrovský M, Hlavinka P, Balek J, Eckersten H, Cloppet E, Calanca P, Gobin A, Vučetić V, Nejedlik P, Kumar S, Lalic B, Mestre A, Rossi F, Kozyra J, Alexandrov V, Semerádová D, Žalud Z (2011). Agroclimatic conditions in Europe under climate change. Glob Change Biol.

[CR36] Ustrnul Z, Wypych A, Winkler JA, Czekierda D (2014) Late spring freezes in Poland in relation to atmospheric circulation. Quaestiones Geographicae 33(3):165–172

[CR37] Ustrnul Z, Wypych A, Kosowski M (2012). Extreme temperatures and precipitation in Poland—an evaluation attempt. Meteorol Z.

[CR38] von Storch H, Zwiers RW (2003) Statistical analysis in climate research. Cambridge University Press. Cambridge

[CR39] Wielgolaski F-E (1999). Starting dates and basic temperatures in phenological observations of plants. Int J Biometeorol.

[CR40] Winkler JA, Andresen JA, Guentchev G, Kriegel RD (2002). Possible impacts of projected temperature change on commercial fruit production in the Great Lakes region. J Great Lakes Res.

[CR41] Worthington C, Hutchinson C (2005). Accumulated growing degree days as a model to determine key developmental stages and evaluate yield and quality of potato in Northeast Florida. Proc Fla State Hort Soc.

[CR42] Wypych A, Ustrnul Z (2011). Spatial differentiation of the climatic water balance in Poland. Idójaras.

[CR43] Yang S, Logan J, Coffey DL (1995). Mathematical formulae for calculating the base temperature for growing degree days. Agric For Meteorol.

[CR44] Zavalloni C, Andresen JA, Flore JA (2006). Phenological models of flower bud stages and fruit growth of “Montmorency” sour cherry based on growing degree-day accumulation. J Amer Soc Hort Sci.

[CR45] Żmudzka E (2004). Climatic background of the agricultural production in Poland in the second half of 20th century. Acta Agrophysica.

[CR46] Żmudzka E (2012). Long term changes of the thermal resources in the vegetative period and the active growth of plants in Poland. Water Environ Rural Areas.

[CR47] Żmudzka E (2013). The influence of circulation patterns on extreme thermal resources in the growing season and the period of active plant growth in Poland (1951–2006). Meteorol Z.

